# The influence of diabetes on short-term outcome following a prosthetic above-the-knee femoro-popliteal bypass

**Published:** 2009-05

**Authors:** TV Mulaudzi, JV Robbs, N Paruk, B Pillay, TE Madiba, V Govindsamy

**Affiliations:** Durban Metropolitan Vascular Service and Nelson R Mandela School of Medicine, University of KwaZulu-Natal, Durban, South Africa; Durban Metropolitan Vascular Service and Nelson R Mandela School of Medicine, University of KwaZulu-Natal, Durban, South Africa; Durban Metropolitan Vascular Service and Nelson R Mandela School of Medicine, University of KwaZulu-Natal, Durban, South Africa; Durban Metropolitan Vascular Service and Nelson R Mandela School of Medicine, University of KwaZulu-Natal, Durban, South Africa; Nelson R Mandela School of Medicine, University of KwaZulu-Natal, Durban, South Africa; Nelson R Mandela School of Medicine, University of KwaZulu-Natal, Durban, South Africa

## Abstract

**Objectives:**

To assess the influence of diabetes mellitus on early morbidity and mortality following a femoro-popliteal bypass.

**Methods:**

Clinical data on patients subjected to a prosthetic above-the-knee femoro-popliteal bypass for atherothrombotic disease over a four-year period in the Durban Metropolitan Vascular Service were culled from a prospectively maintained computerised database. The patients were divided into two groups, diabetic and non-diabetic.

**Results:**

Two hundred and seventeen patient records were analysed; 102 (47%) patients were diabetic and 115 (53%) non-diabetic. The mean age in the two groups was almost similar. Differences noted between the two groups were that there was a higher prevalence of males and cigarette smokers in the non-diabetic group and hypertension among the diabetics. The prevalence of ischaemic heart disease in the two groups was not statistically significant. The majority of patients in both groups presented with critical limb ischaemia.

Overall, 208 (96%) of the patients had their procedures performed using loco regional anaesthesia. The incidence of superficial wound infection between the two groups was not statistically significant. Deep infection, which necessitated removal of the graft, and cardiovascular complications were significantly higher in the diabetics. Four patients (3.9%) in the diabetic group and only one (0.9%) in the non-diabetic group died.

**Conclusion:**

Diabetes mellitus significantly increases the incidence of graft sepsis and cardiovascular morbidity in patients undergoing above-the-knee femoro-popliteal bypass.

## Summary

Patients with peripheral arterial disease (PAD) are at increased risk of having concomitant coronary artery disease (CAD), and the incidence is even higher in those who are diabetic.^1^-^5^ Myocardial disease is the commonest cause of morbidity and mortality in patients with PAD.^6^-^13^ Cardiovascular morbidity and mortality is proportional to the severity of the PAD.^14^ Some of these patients will have occult CAD. Non-invasive cardiac evaluation is able to identify most of these patients, but controversy still exists as to the type of cardiac evaluation to be performed before surgery.^16^,^17^

Antithrombotic medication has been shown to reduce the morbidity and mortality from myocardial complications in patient with PAD.^12^,^13^ Tight blood pressure control, and the use of β-blockers and statins have also been found to reduce the cardiac complication rate post peripheral bypass.^4^-^7^,^11^,^12^,^17^

There is a general perception that diabetic patients do not do as well as non-diabetics after a vascular procedure, but there is little evidence of this. Our study was performed to assess the influence of diabetes mellitus on the results of surgery in patients during the early postoperative phase following an above-the-knee prosthetic femoro-popliteal bypass.

## Patients and methods

Clinical data on patients subjected to a prosthetic above-the-knee femoro-popliteal bypass over a four-year period (2001–2005) in the Durban Metropolitan Vascular Service were culled from a prospectively maintained computerised database. The study included only patients with atherothrombotic disease. In every case the distal anastamosis was made to the proximal popliteal artery above the knee joint.

The patients were divided into two groups, diabetic and nondiabetic. Patients who were known to be diabetic were already on treatment when referred to us and all other patients were evaluated for diabetes mellitus by means of blood tests.

Clinical information related to presenting symptoms, risk factors for atherothrombotic disease as well as co-morbidities were analysed. Thirty-day morbidity was analysed in terms of systemic and local complications as well as mortality in each group.

Statistical analysis was done using the Fishers exact test (two tailed). A *p*-value of < 0.05 was regarded as statistically significant.

## Results

Two hundred and seventeen patient records were analysed; 102 (47%) were diabetic and 115 (53%) were non-diabetic. Table 1 shows the racial composition of the cohort. There was a significantly higher incidence of diabetes mellitus among Indian patients.

**Table 1:** Racial Composition

	*Diabetics*	*Non-diabetics*
Total	102	115
Africans (%)	21 (21)	45 (39)
Indians (%)	68 (67)	40 (35)
Mixed races (%)	1 (1)	2 (2)
Caucasians (%)	12 (12)	28 (24)

Table 2 summarises the patient profile and co-morbidities. The mean age between the two groups was almost similar. Differences noted between the two groups were that there was a higher prevalence of males (*p* = 0.005) in the non-diabetic group as well as a significantly higher proportion of cigarette smokers (*p* = 0.003) in this group. There was a higher prevalence of hypertension (*p* = 0.001) among diabetics. Even though ischaemic heart disease was more prevalent in diabetics, this was found not to be statistically significant (*p* = 0.064).

**Table 2:** Patient Profile And Co-Morbidities

	*Diabetics*	*Non-diabetics*	p*-value*
Total (%)	102 (47)	115 (53)	
Male (%)	56 (55)	85 (74)	0.005
Female (%)	46 (45)	30 (26)	0.005
Age mean (years)	65	63	
Hypertension (%)	54 (53)	35 (30)	0.001
Smokers (%)	68 (67)	101 (89)	0.0003
Hyperlipidaemia (%)	8 (8)	6 (5)	0.61
IHD (%)	23 (23)	14 (12)	0.064
COPD (%)	1 (0.9)	6 (5)	0.17
Lung carcinoma (%)	4	0	0.10

IHD: ischaemic heart disease, CABG: coronary artery bypass graft, COPD: chronic obstructive pulmonary disease.

Clinical presentation is summarised in Table 3. The majority of patients in both groups (81% of diabetic patients and 73% of non-diabetics) presented with critical ischaemia, either with pain at rest or an ischaemic lesion such as an ulcer or gangrenous digits. Fifty-two (51%) of the diabetics had an infected ulcer or gangrene providing a septic focus, as opposed to 45 (39%) of the non-diabetics. This was not statistically significant (*p* = 0.100).

**Table 3:** Clinical Presentation

	*Diabetics*	*Non-diabetics*	p*-value*
Total	102	115	
Disabling claudication (%)	19 (19)	31 (27)	0.20
Resting pain (%)	31 (30)	39 (34)	0.68
Ulcer (%)	33 (32)	15 (13)	0.001
Gangrene (%)	19 (19)	30 (26)	0.25

Overall, 208 (96%) of the patients had their procedures performed using loco regional anaesthesia. Table 4 summarises the complications occurring within 30 days of operation. Wound infection treated by systemic antibiotics and topical dressings occurred in six (5.8%) of the diabetics and 11 (9.6%) of the nondiabetics, but the difference was not significant (*p* = 0.60). There was no relationship between the incidence of wound infection and the presence of an infected foot lesion at presentation (*p* = 0.31).

**Table 4:** Complications Within 30 Days Of Femoro-Popliteal Bypass

	*Diabetics*	*Non-diabetics*	p*-value*
Total	102	115	
Wound sepsis (%)	6 (5.8)	11 (9.7)	0.60
Graft sepsis (%)	6 (5.8)	1 (0.9)	0.05
Cardiovascular complications (%)	7 (6.9)	1 (0.9)	0.03
Died (%)	4 (3.9)	1 (0.9)	0.19

Deep infection involving the graft occurred in six diabetics (5.8%) as opposed to one non-diabetic (0.9%), which was statistically significant (*p* = 0.05). This necessitated the removal of the graft in all patients. It should be noted that in only two of the total seven patients with graft sepsis was there an open, infected lesion on the foot at the time of the bypass. All patients were put on prophylactic antibiotics for 48 hours at the time of surgery.

The commonest systemic complication in the diabetic group involved the cardiovascular system (6.9%); myocardial infarction in five and stroke in two, as opposed to only one (0.9%) cardiovascular complication (myocardial infarction) in the non-diabetic group. This difference between the two groups was significant (*p* = 0.03). All those with cardiovascular complications were Indian patients and had presented with critical limb ischaemia. Four (3.9%) of these patients with cardiovascular complications in the diabetic group were hypertensive and the only patient in the non-diabetic group was also hypertensive. This was not statistically significant (*p* = 0.20). These patients had presented with asymptomatic coronary artery disease.

Four patients (3.9%) in the diabetic group died, two following a myocardial infarction, one following a stroke and one of renal failure. The single death (0.9%) in the non-diabetic group followed a myocardial infarction. This difference was not statistically significant (*p* = 0.19).

## Discussion

There is little to be found in the literature that specifically addresses the influence of diabetes mellitus on peri-operative morbidity, which this study specifically attempts to address in relation to femoro-popliteal bypass. The operative procedure was standardised as far as possible by confining analysis to patients undergoing prosthetic grafting in the above-the-knee position. The overall co-morbidity profile in the two groups of patients was similar, although non-diabetics had a preponderance of males and a much higher incidence of cigarette smoking. There was a higher incidence of hypertension in the diabetics.

In general, diabetics are known to have an increased incidence of septic complications. The patients in this study with wound sepsis were managed with antibiotics and topical chemical debridement, with good results. There was no increased incidence of this type of sepsis in those patients who presented with tissue loss. In addition, the incidence of wound sepsis was not increased in those who were diabetic, whereas there was a significant difference in the incidence of deep sepsis affecting the graft in the diabetics. It is also of interest to note that there was no correlation between deep sepsis and the presence of preoperative tissue loss. As this was a retrospective study, there was a possibility of under reporting, with possibly different results.

Postoperative cardiovascular complications were significantly increased in those who were diabetic. This might have been influenced by the increased incidence of ischaemic heart disease among the diabetics, even though this was found not to be statistically significant. This concurs with the findings of several authors and was mainly due to the increased incidence of associated cardiovascular disease in those diabetics who presented with peripheral arterial occlusive disease.^1^-^5^ As the majority of the patients in this study had critical ischaemia, the incidence of cardiovascular disease would have been expected to be even higher, as its incidence is increased with the severity of peripheral arterial disease.^14^ Of interest is that all patients who had cardiovascular complications were Indians. This was probably due to the fact that there is a higher prevalence of diabetes in this population.

Overall in this series, those who developed myocardial infarction did not have symptomatic coronary artery disease. This calls into question how aggressive one needs to be in the pre-operative workup. There remains no consensus but most authors suggest that only those who have symptomatic coronary artery disease should be referred for cardiological assessment. Others have shown that routine extensive cardiac assessment for patients presenting with peripheral arterial disease does not reduce the morbidity and mortality rate.^15^,^16^

It is our practice to refer patients with symptomatic coronary artery disease for cardiological assessment. What we do not know is whether we should be more aggressive with our Indian diabetic patients as they have demonstrated a higher incidence of cardiovascular complications even though they had been asymptomatic at presentation. We are continuing the study of this subgroup of patients to determine how we can reduce the morbidity and mortality rate.

Strong evidence is emerging in support of beta-blockade and statin administration to reduce the incidence of peri-operative cardiovascular complications.^4^-^8^,^11^,^12^,^17^ Few patients in this study were on beta-blockers and statins when referred to us. Hopefully, the importance of this will be realised and more patients will be placed on this therapy.

Even though this was not statistically significant, all five patients who died had presented with critical ischaemia. Four of these patients died from cardiovascular-related complications and they were all diabetic. This confirms the increased incidence of cardiovascular complications in diabetics and in those with critical ischaemia.^6^-^14^ It is important then for these patients with PAD to have a proper clinical history and examination to identify those with suspected significant coronary artery disease. This group of patients will require further assessment by cardiologists.

## Conclusion

Despite the relatively small patient number in this study, diabetes mellitus was shown to significantly increase the incidence of graft sepsis and cardiovascular morbidity in patients undergoing above-the-knee prosthetic bypass for femoro-popliteal occlusive disease.
